# Eradication of intracellular *Francisella tularensis *in THP-1 human macrophages with a novel autophagy inducing agent

**DOI:** 10.1186/1423-0127-16-110

**Published:** 2009-12-09

**Authors:** Hao-Chieh Chiu, Shilpa Soni, Samuel K Kulp, Heather Curry, Dasheng Wang, John S Gunn, Larry S Schlesinger, Ching-Shih Chen

**Affiliations:** 1 Division of Medicinal Chemistry and Pharmacognosy, College of Pharmacy, The Ohio State University, Columbus, OH 43210, USA; 2 Center for Microbial Interface Biology, The Ohio State University, Columbus, OH 43210, USA; 3 Department of Molecular Virology, Immunology and Medical Genetics, The Ohio State University, Columbus, OH 43210, USA; 4 Division of Infectious Diseases, Department of Internal Medicine, The Ohio State University, Columbus, OH 43210, USA

## Abstract

**Background:**

Autophagy has been shown recently to play an important role in the intracellular survival of several pathogenic bacteria. In this study, we investigated the effect of a novel small-molecule autophagy-inducing agent, AR-12, on the survival of *Francisella tularensis*, the causative bacterium of tularemia in humans and a potential bioterrorism agent, in macrophages.

**Methods and results:**

Our results show that AR-12 induces autophagy in THP-1 macrophages, as indicated by increased autophagosome formation, and potently inhibits the intracellular survival of *F. tularensis *(type A strain, Schu S4) and *F. novicida *in macrophages in association with increased bacterial co-localization with autophagosomes. The effect of AR-12 on intracellular *F. novicida *was fully reversed in the presence of the autophagy inhibitor, 3-methyl adenine or the lysosome inhibitor, chloroquine. Intracellular *F. novicida *were not susceptible to the inhibitory activity of AR-12 added at 12 h post-infection in THP-1 macrophages, and this lack of susceptibility was independent of the intracellular location of bacteria.

**Conclusion:**

Together, AR-12 represents a proof-of-principle that intracellular *F. tularensis *can be eradicated by small-molecule agents that target innate immunity.

## Background

Macroautophagy (called autophagy hereafter) is a cellular response of eukaryotic cells to a number of deleterious stimuli including nutrient deprivation, organelle damage and accumulation of unfolded proteins [1]. In addition, evidence indicates that autophagy also aids in controlling infection by certain microorganisms, including viruses, bacteria and parasites [2]. Indeed, the induction of autophagy by amino acid starvation, interferons or pharmacological agents has been shown to decrease the survival of various intracellular bacteria, including *Mycobacterium tuberculosis*, Group A *Streptococcus pyrogenes *and *Salmonella typhimurium *[3-5]. Thus, the induction of autophagy may represent a viable therapeutic approach for the treatment of infections caused by intracellular bacteria that is worthy of further investigation.

*Francisella tularensis *is a Gram negative, facultative coccobacillus that causes the zoonotic disease, tularemia [6,7]. Depending on the route of infection, *F. tularensis *can lead to different forms of tularemia. Inhalation of bacteria causes the most severe form of the disease, pneumonic tularemia, which has a mortality rate as high as 60 percent in the absence of appropriate treatment [6-8]. Because of the potential to inflict severe disease in a large number of people with an aerosolized form of the bacteria, *F. tularensis *is classified in Category A of potential biological warfare agents by the U.S. Centers for Disease Control and Prevention [9,10]. Moreover, in the absence of an U.S. Food and Drug Administration-approved vaccine and in light of the potential existence of antibiotic-resistant strains of *F. tularensis *created in the early 1990s [9-11], the development of new antibacterial agents with novel mechanisms against *F. tularensis *has become a priority for public safety.

In infected hosts, *F. tularensis *is primarily found in the macrophage [8]. After entry into macrophages by phagocytosis, *F. tularensis *blocks the fusion of *Francisella*-containing phagosomes with lysosomes and later escapes into the cytosol where it proliferates to a high number [12-14]. Subsequently, *F. tularensis *induces infected host cells to undergo apoptosis or pyroptosis, which leads to release of bacteria and infection of new cells [15,16]. In addition to this cytosolic proliferation and induction of host cell death, intracellular *F. tularensis *have also been found to reside in *Francisella*-containing vacuoles (FCVs) at later stages of intracellular infection. The FCVs are double-membraned vacuoles, which microscopically appear similar to the autophagosmes formed during autophagy [17,18]. Blockage of autophagy decreased the colocalization of intracellular *F. tularensis *with FCVs in infected host cells [17,19]. Moreover, *F. tularensis *mutants which are incapable of escaping from phagosomes were found to be surrounded by autophagosome-like vacuoles at the early stage of intracellular infection, suggesting that autophagy could play an important role in controlling intracellular growth of *Francisella *within phagosomes [18,19].

In this study, we demonstrate that AR-12 (a.k.a. OSU-03012), a novel small-molecule autophagy-inducing agent, is able to eradicate intracellular *F. tularensis ssp. novicida *(called *F. novicida *hereafter) and *F. tularensis *(type A strain Schu S4) without causing cytotoxicity to the host cells. Furthermore, inhibition of autophagosome formation and lysosomal degradation completely reversed this AR-12-induced killing of intracellular *F. tularensis*, indicating that the anti-*Francisella *activity of this agent is mediated primarily through an autophagy-dependent mechanism. Together, our findings show that induction of autophagy is an effective approach for the control of intracellular *F. tularensis *in macrophages and suggest that AR-12 can serve as a scaffold for the development of more potent autophagy-inducing antibacterial agents.

## Materials and methods

### Bacteria

*F. novicida *strain U112 and *F. tularensis *strain Schu S4 (type A) were used throughout this study. Construction of a quadruplicate acid phosphatase mutant strain (JSG2871) of *F. novicida *was described in a previous study [18]. Experiments involving Schu S4 were conducted in a CDC select agent-approved BSL-3 laboratory at The Ohio State University. Bacteria were grown at 37°C on chocolate II agar (Becton, Dickinson and Company, Franklin Lakes, NJ) or in tryptic soy broth (TSB; Becton, Dickinson and Company) supplemented with 0.025% (w/v) iron (III) pyrophosphate (Sigma-Aldrich, St. Louis, MO) and 0.1% (w/v) cysteine hydrochloride (MP Biomedicals, Solon, OH).

### Macrophages

The THP-1 human monocytic leukemia cell line was maintained in RPMI 1640 medium (GIBCO-BRL, Invitrogen Corp., Carlsbad, CA) containing 10% fetal bovine serum (FBS; GIBCO-BRL). To induce differentiation, THP-1 cells were treated with 20 nM 12-O-tetradecanoylphorbol 13-acetate (TPA; Sigma-Aldrich, St. Louis, MO) for 48 h. Cells were washed twice with pre-warmed PBS and cultured with fresh RPMI 1640 medium containing 10% FBS at 37°C in a humidified incubator containing 5% CO_2 _prior to experimentation.

### Reagents and antibodies

AR-12 was synthesized in-house as described previously with purity exceeding 99% as shown by nuclear magnetic resonance spectroscopy (300 MHz) [20]. 3-Methyl adenine (3-MA) and chloroquine were obtained from Sigma-Aldrich. Stock solutions of AR-12 and chloroquine were prepared in DMSO and diluted in culture medium for treatment of cells (final concentration of DMSO, 0.1%). 3-MA was directly dissolved in cell culture medium and freshly prepared before each experiment. The following antibodies were used in this study: anti-LC3 and anti-LC3 II (MBL, Woburn, MA); anti-*Francisella tularensis ssp. novicida *(ImmunoPrecise, Victoria, BC); Alexa Red-conjugated goat anti-mouse IgG and FITC-conjugated goat anti-mouse IgG (Invitrogen, Carlsbad, CA).

### Assay for intracellular survival of *Francisella *in macrophages

*F. novicida *and *F. tularensis *(type A strain Schu S4) grown overnight on chocolate II agar plates were suspended in PBS to a concentration of approximately 10^10 ^CFU/ml (as estimated by an O.D. of 1.0 at 600 nm). To facilitate bacterial uptake by macrophages, bacteria were opsonized with human complement by incubating in RPMI-1640 medium containing 10% normal human serum for 30 min at 37°C with agitation [21]. Bacteria were added at a MOI of 50 to TPA-differentiated THP-1 macrophages seeded in 24-well plates at 2.5 × 10^5 ^cells/well [18]. Plates were cultured with rocking for 30 min at 37°C in a humidified incubator containing 5% CO_2_, and then incubated for an additional 1.5 h. Bacteria and macrophages were exposed to 50 μg/ml of gentamicin for 30 min, followed by two washes with pre-warmed PBS to remove killed extracellular bacteria [15]. Infected macrophages were then treated in triplicate with various concentrations of AR-12 for 3 h, after which culture medium was collected from each well and macrophages lysed with 500 μl of 0.1% sodium deoxycholate in PBS at 37°C for 5 min to release intracellular bacteria [14]. Bacteria present in the collected culture medium, either as free bacteria or within unattached macrophages, were harvested by centrifugation at 16,000 × g for 5 min, followed by resuspension in the macrophage lysates obtained previously. Cell lysates were serially diluted with PBS and spread onto agar plates supplemented with 0.025% (w/v) iron (III) pyrophosphate and 0.1% (w/v) cysteine hydrochloride, or chocolate II agar plates. CFU were calculated after incubation for 24 h at 37°C. Survival of intracellular bacteria in drug-treated macrophages was calculated as a percentage of that in control (untreated) cells.

### Immunofluorescence microscopy

To visualize intracellular *F. novicida*, bacteria were transformed with a green fluorescent protein (GFP) expressing plasmid (pKK214) as described in a previous study [22]. Infection of THP-1 macrophages with GFP-labeled *F. novicida *was performed as described above for unmodified *F. novicida*. After treatment with vehicle or AR-12, cells were washed three times with cold PBS, fixed with 4% formaldehyde (Sigma) in PBS for 20 min at 25°C, and then permeabilized with 0.5% Triton X-100 in PBS for 15 min followed by blocking with 3% bovine serum albumin (BSA) in PBS overnight at 4°C. After three washes with PBS, infected macrophages were incubated with primary antibody in PBS containing 1% BSA for 1 h at 25°C and then with Alexa Red- or FITC-conjugated secondary antibody for 1 h at 25°C. Macrophage nuclei were stained with 4'-6-diamidino-2-phenylindole (DAPI) contained in the Vectashield Mounting Medium (Vector Laboratories, Burlingame, CA). The slides were examined using a Nikon TE300 wide-field fluorescent microscope equipped with a digital camera (CoolSnap HQ, Roper Scientific, Tucson, AZ) or a Zeiss LSM 510 confocal laser scanning microscope system. To assess co-localization of intracellular bacteria with autophagosomes, three-dimensional images acquired by confocal microscopy were utilized to ensure the direct contact of bacteria with autophagosomes.

### Immunoblotting

Cells were washed with cold PBS, then suspended in M-PER protein extraction reagent (Pierce, 7 Rockford, IL), vortex vigorously and incubated on ice for 10 min. After centrifugation at 14,000 × *g *for 15 min at 4°C, the lysate supernatants were mixed with 4× Laemmli buffer and incubated at 95°C for 10 min. Equivalent amounts of total protein were resolved on a SDS-acrylamide gel (20 μg/lane) and transferred to 0.2 μm nitrocellulose membranes (Gelman, Pall Corp., East Hills, NY). The membranes were blocked with 3% skim milk in TBS for 30 min and then washed twice with 0.5% Tween-20 in TBS (TBST). The membrane was incubated with primary antibody at the appropriate dilution in TBST for 12 h at 4°C, washed three times with TBST, incubated with HRP-conjugated goat IgG secondary antibody in TBST containing 1% skim milk for 2 h, and then washed three times with TBST. The immunopositive bands were visualized by enhanced chemiluminescence (GE Amersham, Piscataway, NJ]) followed by exposure of X-ray film (Hyperfilm, GE Amersham). Quantification of the density of bands was performed using Gel-Pro Analyzer (V3.1, Media Cybernetics, Bethesda, MD).

### Macrophage viability assay

The effect of AR-12 on macrophage viability was assessed by using the 3-(4,5-dimethylthiazol-2-yl)-2,5-diphenyltetrazolium (MTT) assay [23]. THP-1 macrophages were seeded into 96-well plates at 2.5 × 10^4 ^cells/well (minimum of six wells per test group) in RPMI 1640 medium supplemented with 10% FBS, and then incubated overnight at 37°C in a humidified incubator containing 5% CO_2_. The medium from each well was removed and replaced with fresh 10% FBS-supplemented RPMI 1640 medium containing various concentrations of AR-12. Controls received DMSO alone at a concentration equal to that in drug-treated cells. After 3 h of treatment, the medium was removed, replaced by 100 μl of 0.5 mg/ml of MTT in 10% FBS-containing medium, and the cells were incubated at 37°C for 30 min. Medium was removed from each well, and the reduced MTT dye was solubilized in 100 μl/well of DMSO. Absorbance at 570 nm was determined on a plate reader. The viability of drug-treated cells was calculated as a percentage of vehicle-treated control cells, and an IC_50 _for cell viability was determined by using CalcuSyn software (Biosoft, Cambridge, UK). In addition to the MTT assay, the effect of AR-12 on the viability of *F. novicida*-infected THP-1 macrophages was confirmed by the lactate dehydrogenase (LDH) release assay [24] using the CytoTox 96^® ^Non-Radioactive Cytotoxicity Assay kit (Promega, Madison, WI, USA).

### Extracellular bacterial growth assay

*F. novicida *grown overnight on a chocolate II agar plate were suspended in PBS to an O.D. of 1.0 at 600 nm, which was equivalent to 10^10 ^CFU/ml, and then diluted in modified TSB to a final concentration of 10^4 ^CFU/ml. The bacterial suspension was exposed to various concentrations of AR-12 in triplicate in 96-well plates. Bacterial growth in each well was monitored spectrophotometrically on a microplate reader (Molecular Devices, Sunnyvale, CA) at 37°C and a wavelength of 600 nm with readings taken every 30 min for 8 h.

### Statistical analysis

Data are expressed as means ± SD. Group means were compared using a two-tailed *t*-test for independent samples. Differences were considered significant at *P *< 0.05. Statistical analyses were performed using SPSS for Windows (Version 16.0; SPSS, Inc. Chicago, IL).

## Results

### AR-12 induces autophagy in human macrophages without causing cytotoxicity

**Figure 1:**
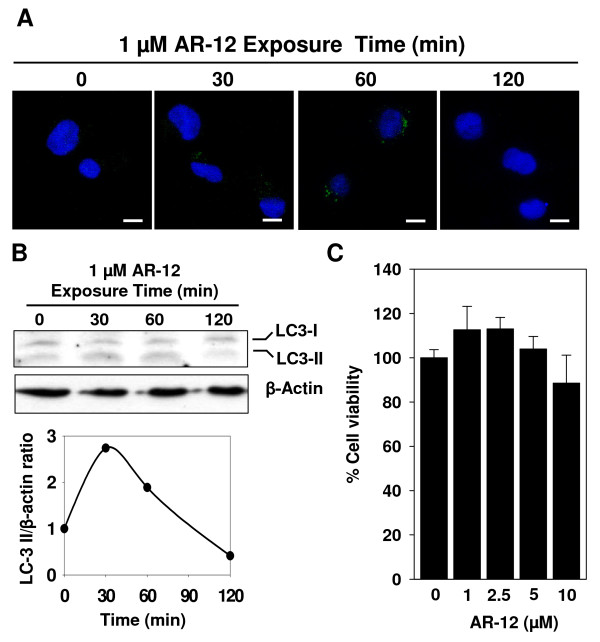
**Induction of autophagy in THP-1 macrophages by AR-12**. (A) AR-12 induced autophagosome formation in uninfected THP-1 macrophages. THP-1 cells were induced to differentiate by treatment with 12-O-tetradecanoylphorbol 13-acetate and then treated as indicated. Autophagosomes were visualized by immunofluorescence microscopy after staining with antibody against LC3-II. Scale bar, 10 μm. (B) AR-12 induced the accumulation of LC3-II in uninfected THP-1 macrophages. *Above*: Western blot analysis of the time-dependent effect of AR-12 on LC3-II formation in uninfected THP-1 macrophages. β-Actin was used as a loading control. *Below*: The ratio of LC3-II/β-actin in the blot after different time of AR-12 exposure (C) AR-12 did not affect the viability of uninfected THP-1 macrophages. Cells were treated with the indicated doses of AR-12 for 3 h and cell viability was assessed by the MTT assay. *Points*, mean; *bars*, ± SD (n = 6).

AR-12 is an orally bioavailable small-molecule inhibitor of phosphoinositide-dependent kinase (PDK)-1 that was derived by structural modification of the cyclooxygenase-2 (COX-2) inhibitor, celecoxib, but is devoid of COX-2-inhibitory activity [20]. In addition to PDK-1 inhibition, recent findings indicate that AR-12 is capable of inducing autophagy in a variety of cells at concentrations of 1 - 5 μM [25-27]. Importantly, we recently showed that AR-12 caused the clearance of intracellular *Salmonella *Typhimurium, in part, through this autophagy-inducing activity [28]. To assess whether AR-12 can induce autophagy in human macrophages, THP-1 macrophages were exposed to 1 μM of AR-12, and then evaluated for evidence of autophagy by immunocytochemistry and Western blot analysis. First, the formation of autophagosomes in the cytosol was visualized by immunofluorescent staining with antibody against microtubule-associated protein light chain 3-II (LC3-II), a specific autophagosome marker. During autophagy, the cytoplasmic form of the protein, LC3-I, is cleaved and recruited to the autophagophore, where LC3-II is generated via site-specific lipidation. As shown in Fig. 1A, AR-12 induced a transient increase in the number and size of autophagosomes in the cytosol of human macrophages that peaked at 60 min after drug exposure. Second, immunoblotting of LC3 protein in AR-12-treated macrophages also showed a transient increase in LC3-II levels (Fig. 1B). This fluctuation in the presence of autophagosomes and LC3-II in AR-12-treated macrophages indicated that autophagy was activated and that the subsequent lysosomal degradation of autophagosomes was unaffected. Although excessive autophagic activity can lead to cell death, no cytotoxic effect of AR-12 on THP-1 macrophages was observed after 3 h of treatment with concentrations of up to 10 μM (Fig. 1C). Together, these findings indicate AR-12 is capable of inducing autophagy in THP-1 macrophages at a concentration that exhibits no cytotoxicity.

### AR-12 inhibits intracellular survival of *Francisella *tularensis in human macrophages

**Figure 2:**
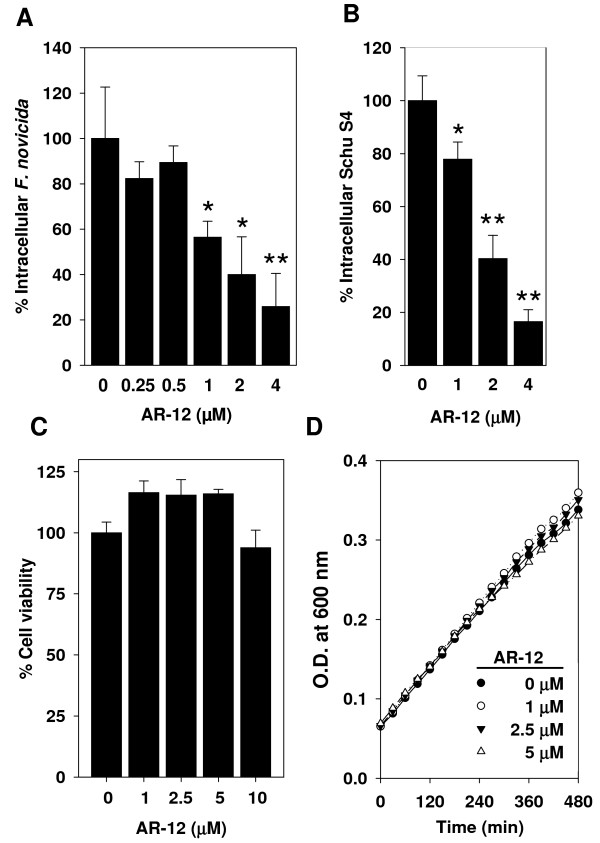
**Inhibition of intracellular survival of *F. tularensis *in THP-1 macrophages by AR-12**. (A) Dose-dependent effect of AR-12 (3 h treatment) on the intracellular survival of *F. novicida *in THP-1 macrophages. Post-treatment CFU values are expressed as a percentage of those in the control group. *Columns*, mean; *bars*, ± SD (n = 3). *P < 0.05, **P < 0.01. (B) Dose-dependent effect of AR-12 (3 h treatment) on the intracellular survival of *F. tularensis *(a type A strain, Schu S4) in THP-1 macrophages. *Points*, mean; *bars*, ± SD (n = 3). *P < 0.05, **P < 0.01. (C) AR-12 did not show cytotoxic effect on the viability of *F. novicida *infected THP-1 macrophages. Cells infected with *F. novicida *and treated with the indicated doses of AR-12 for 3 h. Cell viability was assessed by the MTT assay. *Points*, mean; *bars*, ± SD (n = 3). (D) Effect of AR-12 on the growth of *F. novicida *in modified TSB broth. *Points*, mean; *bars*, ± SD (n = 6).

To examine whether the autophagy induced by AR-12 is associated with an increase in colocalization of intracellular *F. novicida *with autophagosomes, cells infected with GFP-labeled *F. novicida *were treated with vehicle or 1 μM of AR-12 for 60 min and then probed with LC3-II antibody. The colocalization of bacteria with LC3-II-positive puncta was determined by confocal fluorescence microscopy. Data indicated that approximately 15% of *F. novicida *in AR-12-treated THP-1 macrophages were co-localized with LC3-II-positive puncta, compared to 5% in vehicle-treated macrophages after 1 h of AR-12 treatment (data not shown). To examine whether this increase in bacteria-autophagosome colocalization is associated with reductions in the intracellular survival of *F. novicida*, infected THP-1 macrophages were exposed to different concentrations of AR-12 for 3 h, and then the number of surviving intracellular bacteria was assessed by CFU counts. As shown in Fig. 2A, the survival of intracellular *F. novicida *was significantly reduced by AR-12 at concentrations ≥1 μM. As AR-12 has not been shown to induce autophagy at sub-μM concentrations, these data indicate a correlation between autophagy induction and inhibition of intracellular bacterial survival in AR-12-treated THP-1 macrophages. In addition to *F. novicida*, the effect of AR-12 on intracellular *F. tularensis *(type A strain Schu S4) was also examined. With exposure to various concentrations of AR-12 for 3 h, the survival of intracellular Schu S4 in THP-1 macrophages decreased in a dose-dependent manner (Fig. 2B). This AR-12-induced intracellular bacterial killing, however, was not attributable to the death of infected host cells since AR-12 had no appreciable effect on the viability of bacteria-infected THP-1 macrophages after 3 h of treatment with concentrations up to 10 μM (Fig. 2C). A similar lack of cytotoxicity was also observed at 6 and 12 h of treatment with 5 μM of AR-12 (data not shown). Confirmatory assays using LDH release as an indicator of cytotoxicity revealed a similar lack of effect on the viability of infected THP-1 macrophages (data not shown). Furthermore, to determine whether this AR-12-induced clearance of intracellular *Francisella *from macrophages involved a direct action of the drug on bacteria, *F. novicida *were exposed to AR-12 during growth in modified TSB. Figure 2D shows that AR-12 exhibited no direct inhibitory effect on the growth of *F. novicida*, suggesting that the inhibition of intracellular survival of *Francisella *is mediated indirectly via effects on host cells. Together, these findings show that AR-12 is capable of inhibiting growth of both human-virulent and human-avirulent subspecies of *F. tularensis *in human macrophages through a host cell-directed mechanism.

### The inhibitory activity of AR-12 against intracellular *Francisella *is autophagy-dependent

**Figure 3:**
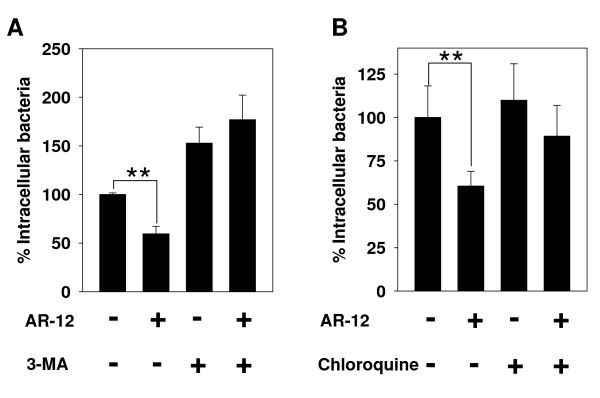
**Inhibition of the intracellular survival of *F. novicida *in THP-1 macrophages by AR-12 involves an autophagy-dependent mechanism**. (A) The autophagy inhibitor 3-MA prevented AR-12-induced inhibition of intracellular bacterial survival. THP-1 macrophages were infected with *F. novicida *and then treated for 3 h with 2 μM AR-12 or DMSO in the presence and absence of 10 mM 3-MA as indicated. The number of surviving intracellular bacteria was determined and expressed as a percentage of that in the DMSO only group. *Points*, mean; *bars*, ± SD (n = 3). **P < 0.01. (B) Chloroquine, an inhibitor of lysosomal degradation, prevented AR-12-induced inhibition of intracellular bacterial survival. THP-1 macrophages were infected with *F. novicida *and then treated for 3 h with 2 μM AR-12 or DMSO in the presence and absence of 10 μM chloroquine as indicated. The number of surviving intracellular bacteria was determined and expressed as a percentage of that in the DMSO only group. *Points*, mean; *bars*, ± SD (n = 3). **P < 0.01.

To verify the role of autophagy in AR-12-induced killing of intracellular *Francisella*, we assessed the effect of blocking autophagy on the *anti-Francisella *activity of AR-12. 3-MA suppresses autophagy through inhibition of class III phosphatidylinositol-3-kinase (PI3K), the enzyme that catalyzes the production of phosphoinositide-3-phosphate, which plays an important role in the formation of autophagosomes. Treatment of infected THP-1 macrophages with 3-MA reversed the inhibitory effect of AR-12 on the survival of intracellular *F. novicida *(Fig. 3A).

At late stages of the autophagic process, autophagosomes fuse with lysosomes to form autolysosomes, the contents of which are subsequently degraded by lysosomal enzymes. Consequently, to examine whether the blockage of lysosomal degradation will affect AR-12's anti-*Francisella *activity, we utilized chloroquine, an inhibitor of lysosomal degradation, to block the degradation of autophagosomal contents. As shown, the reduction of intracellular *F. novicida *induced by AR-12 was completely reversed in the presence of 10 μM of chloroquine (Fig. 3B). Together, these findings indicated that autophagy plays a major role in the eradication of intracellular *F. tularensis *by AR-12.

### The susceptibility of intracellular *Francisella *to AR-12 varies at different time points post-infection

**Figure 4:**
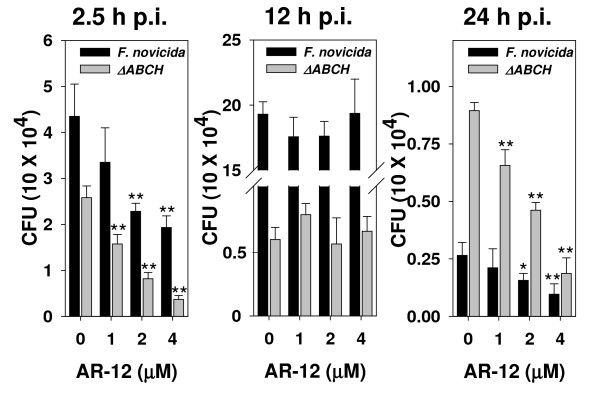
**Susceptibility of intracellular *F. novicida *to AR-12 varies at different time points post-infection**. THP-1 macrophages were infected with *F. novicida *(dark bar) or a quadruplicate acid phosphatase deletion mutant (ΔABCH; gray bar) and treated with various doses of AR-12 for 3 h beginning at different time points post-infection. The number of surviving intracellular bacteria was determined by the CFU assay. *Columns*, mean; *bars*, ± SD (n = 3). *P < 0.05, **P < 0.01.

During the course of intracellular infection of macrophages, *F. tularensis *is located within different cellular compartments including the phagosome from which it escapes, the cytosol where it proliferates, and, at later stages, the FCVs. Mohapatra *et al*. have demonstrated that during infection of THP-1 human macrophages with *F. novicida*, 98% and 60% of intracellular bacteria reside in the vacuoles at 2 h and 24 h post-infection, respectively, while at 12 h post-infection, only about 1% are present in vacuoles as the vast majority of bacteria have escaped to the cytosol [18]. To assess whether the inhibitory activity of AR-12 on the intracellular survival of *F. novicida *varies with the intracellular location of bacteria, infected THP-1 macrophages were treated with various doses of AR-12 for 3 h starting at 2.5, 12 and 24 h post-infection, and the surviving intracellular bacteria were enumerated by the CFU assay. While AR-12 showed potent dose-dependent inhibitory activity on intracellular *F. novicida *at 2.5 h and 24 h post-infection, no significant inhibition of intracellular survival was observed at 12 h post-infection (Fig. 4). This result indicates a lack of inhibitory activity against bacteria at 12 h post-infection, and that AR-12's antibacterial activity might be specific to *Francisella *enclosed within intracellular vacuoles.

To verify whether the intracellular location affects the susceptibility of intracellular *Francisella *to AR-12, the effect of AR-12 on a quadruplicate acid phosphatase deficient strain (ΔABCH) of *F. novicida *was examined. The combined deletion of four acid phosphatases (A, B, C and H) of *F. novicida *has been shown to impair the ability of bacteria to escape the phagosome as these bacteria were demonstrated to always reside in a vacuolar compartment in THP-1 macrophages at 2, 12 and 24 h post-infection [18]. However, as shown in Figure 4, whereas the intracellular ΔABCH mutant was susceptible to AR-12 at 2.5 h and 24 h post-infection, it was resistant at 12 h post-infection comparable to the wild type strain, indicating that the resistance of intracellular *F. novicida *to AR-12 at 12 h post-infection is not due to the intracellular location of bacteria but rather to another mechanism.

## Discussion

Recently, the concept of boosting host defense mechanisms against intracellular pathogens has received wide attention in the infectious disease arena [29]. From a therapeutic perspective, targeting host immunity with an orally bioavailable small-molecule agent represents a novel strategy for antimicrobial therapy. Here we present findings that provide proof-of-principle of the feasibility of treating *Francisella *infection by targeting autophagy in phagocytes with a small-molecule agent. Our results show that AR-12 is a potent inhibitor of the intracellular survival of *F. tularensis *and activator of autophagy in macrophages at concentrations that induce no cytotoxicity in host macrophages. Although AR-12 has been shown to be cytotoxic to cancer cells, the antibacterial effects of AR-12 occur at lower concentrations and after shorter treatment durations. Moreover, it is worth noting that our previous *in vivo *evaluations of AR-12 in murine models of cancer revealed that continuous treatment with AR-12 were well tolerated and induced no dose-limiting toxicities [30,31] in association with plasma concentrations of approximately 2.5 μM (Chen, unpublished data). These findings suggest that toxicities associated with the levels of AR-12 needed for antibacterial effects, if they occur, will be minimal.

Several intracellular bacteria, including *Shigella*, *Legionella *and *Burkholderia*, are capable of evading autophagic eradication by inhibiting the activation of autophagy with bacterial secretory proteins [32-34]. The findings presented here indicate that intracellular *F. tularensis *at 12 h post-infection is not susceptible to the antibacterial activity of AR-12; in contrast to the susceptibility seen at 2.5 h and 24 h post-infection. Since AR-12-induced killing of intracellular *Francisella *is mediated primarily through an autophagy-dependent mechanism, we speculate that bacteria evade the antibacterial effect of AR-12 by inhibiting autophagy activation at 12 h post-infection. This notion is supported by evidence showing that intracellular infection with *Francisella *down-regulates several autophagy-related genes in THP-1 human monocytes, including ATG5, ATG12, ATG16L2, ATG7, ATG4A and class III PI3K [35,36]. Although these reported data were obtained at 24 h post-infection, we cannot exclude the possibility that this down-regulation of autophagy by *Francisella *occurs at an earlier stage of infection such as after phagocytosis and exposure to the phagosomal environment. Moreover, our findings from the use of the quadruplicate acid phosphatase mutant strain (ΔABCH) indicate that this resistance to AR-12 involves pathways affecting autophagy or other aspects of host resistance that are independent of the intracellular location of the organism. Nonetheless, the fact that intracellular *Francisella *are located within autophagosomes at late stages of infection suggest that this autophagy inhibitory activity of the bacteria declines, resulting in restoration of susceptibility to autophagic eradication and rendering them sensitive to the antibacterial activity of AR-12.

AR-12 has been reported to inhibit a variety of cellular enzymes, including PDK-1 and P21 activated kinase-1 (PAK-1), and to induce endoplasmic reticulum (ER) stress [37-39]. Among these activities, the induction of ER stress has been shown to contribute to the AR-12-induced activation of autophagy in cancer cells for which the activity of PKR-like ER kinase (PERK) was determined to be important [26]. Activation of PERK, considered to be the central regulatory kinase for the unfolded protein response in eukaryotic cells, leads to autophagosome formation through the phosphorylation eIF2α and subsequent formation of the Atg5-Atg12(-Atg6) complex [40,41]. Although important for AR-12-induced autophagy in cancer cells, it is not known whether PERK has a similar role in infected macrophages. Moreover, no evidence to date has shown that AR-12 interacts directly with PERK leading to its activation. Indeed, AR-12 has been shown to inhibit the activities of PDF-1 and PAK-1 through interaction with their kinase domains, suggesting that the activation of PERK activity is an indirect effect of the drug. Based on the reported activities of celecoxib, the compound from which AR-12 was derived, other potential targets of AR-12 include carbonic anhydrase, sarcoplasmic/ER calcium ATPase (SERCA), COX-1 and COX-2 [42]. Among these enzymes, SERCA is interesting in that it plays an important role in regulating [Ca^2+^] in the ER. Inhibition of SERCA will cause Ca^2+ ^efflux from ER to the cytosol, leading to the unfolded protein response and subsequent activation of PERK. Although there is no direct evidence showing that AR-12 can interact with SERCA, the cytosolic [Ca^2+^] of cancer cells increases immediately after exposure to AR-12 which suggests that AR-12 may inhibit SERCA activity [31]. Identification of the mechanism of how AR-12 induces autophagy should facilitate the development of more potent and specific host cell-targeted antibacterial agents.

We recently reported that celecoxib, the parent compound of AR-12, also exhibits anti-*Francisella *activity, and that subsequent screening of a celecoxib-based focused compound library identified Compound 20, a novel analog with multi-fold greater antibacterial activity [31]. Unlike AR-12, however, Compound 20 showed direct growth-inhibitory activity against *F. novicida *and *F. tularensis *(Schu S4, a type A strain) at low μM concentrations. While the intracellular growth of *Francisella *was also inhibited by Compound 20, this occurred at concentrations that far exceeded those shown for AR-12. Although the antibacterial target of Compound 20 has not been identified, these differences in activity suggest that Compound 20 and AR-12 represent two novel classes of compound with distinct antibacterial mechanisms. These findings highlight the potential value of existing drugs as important sources of lead compounds for the development of novel antibacterial agents.

## Competing interests

The authors declare that they have no competing interests.

## Authors' contributions

HCC conceived of the study, designed and performed most experiments, and drafted the manuscript. SS conducted the experiment with virulent *Francisella *tularensis strain Schu S4. SKK helped to design experiment and revised the manuscript. HC helped the assay of intracellular *Francisella novicida *in macrophage. DW conducted the synthesis of AR-12. JSG provided the ΔABCH strain of *F. novicida *and helped to design experiments. LSS helped to design experiments and revised the manuscript. CSC conceived of the study and revised the manuscript. All authors read and approved the final manuscript.
